# H/D Exchange Processes in Flavonoids: Kinetics and Mechanistic Investigations

**DOI:** 10.3390/molecules26123544

**Published:** 2021-06-10

**Authors:** Federico Bonaldo, Fulvio Mattivi, Daniele Catorci, Panagiotis Arapitsas, Graziano Guella

**Affiliations:** 1 Bioorganic Chemistry Laboratory, Department of Physics, University of Trento, 38123 Trento, Italy; federico.bonaldo@alumni.unitn.it (F.B.); daniele.catorci@unitn.it (D.C.); 2 Department of Cellular, Computational and Integrative Biology-CIBIO and C3A, University of Trento, 38123 Povo Trento, Italy; fulvio.mattivi@unitn.it; 3 Department of Food Quality and Nutrition, Research and Innovation Centre, Fondazione Edmund Mach (FEM), 38098 San Michele all’Adige, Italy; panagiotis.arapitsas@fmach.it

**Keywords:** H/D isotopic exchange, flavonoids, kinetics, reaction mechanism, NMR spectroscopy

## Abstract

Several classes of flavonoids, such as anthocyanins, flavonols, flavanols, and flavones, undergo a slow H/D exchange on aromatic ring A, leading to full deuteration at positions C(6) and C(8). Within the flavanol class, H-C(6) and H-C(8) of catechin and epicatechin are slowly exchanged in D_2_O to the corresponding deuterated analogues. Even quercetin, a relevant flavonol representative, shows the same behaviour in a D_2_O/DMSOd_6_ 1:1 solution. Detailed kinetic measurements of these H/D exchange processes are here reported by exploiting the time-dependent changes of their peak areas in the ^1^H-NMR spectra taken at different temperatures. A unifying reaction mechanism is also proposed based on our detailed kinetic observations, even taking into account pH and solvent effects. Molecular modelling and QM calculations were also carried out to shed more light on several molecular details of the proposed mechanism.

## 1. Introduction

Flavonoids are polyphenolic compounds that are diverse in both chemical structure and properties. Since flavonoids are naturally present in fruits, vegetable, and tea, they are an integral part of the human diet. It is generally accepted that diary flavonoids have important biological effects [1,2].

**Scheme 1:**
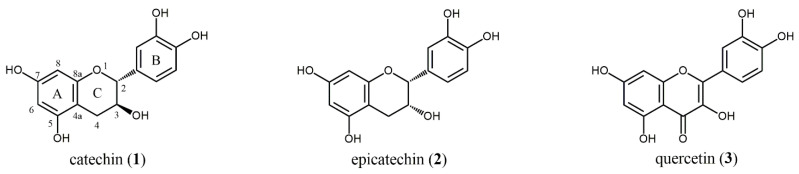
Scheme showing the studied chemical species from flavanol (**1**–**2**) and flavonol (**3**) classes.

Flavonoids often demonstrate phytoalexin and antioxidant properties, important for promoting abiotic and biotic stress tolerance. The antioxidant activity of flavonoids depends upon the arrangement of functional groups on the molecular skeleton [3,4]. Flavonoids are divided into several classes of compounds based on substituents and the functional groups pattern on the shared backbone (Scheme 1).

The key feature that gives flavonoids most of their characteristic properties seems to be an unusually high local concentration of *ortho*-phenolic hydroxyl groups [5]. In later years, great importance has been given to the reactivity of the aromatic rings of flavonoids, due to their peculiar hydroxyl substitution pattern towards other organic and inorganic molecules. Two *o*-hydroxyl groups and an ether-oxygen atom are attached to ring A, in an alternated fashion, and two or more hydroxyl groups are attached to ring B generating a very unique reactivity on the carbon sites of the aromatic rings. Based on polyhydroxyl configuration on the benzene rings of flavonoids, the B ring is considered to be prone to redox processes (catechol to *o*-quinone conversion) [6,7], whilst ring A would be much more reactive to electrophilic substitutions at the strongly activated nucleophilic sites C(6) and C(8). Among the flavonoids, structural changes in the B ring are also considered to influence the antioxidant activity. 

We recently described, within the class of flavan-3-ols, an interesting case of ring C reactivity towards sulfite addition leading to the corresponding 4-sulfonated analogues [8]. During this investigation, we also observed the slow disappearance of the NMR signals of H-C(6) and H-C(8) in ring A when D_2_O was used as a solvent for the flavan-3-ol substrates, evidently due to solvent-mediated H/D exchange at these positions. In fact, during kinetic measurements of sulfonation and thermal-induced isomerization of these flavanols [8], we observed that H/D exchange occurred, within a daytime scale, on both the aromatic protons of ring A.

We were not the first to observe this phenomenon in polyphenols. Several studies already reported on the modification of the H-NMR spectra of different flavonoids in the presence of deuterated solvent [9]. This exchange was first observed in catechin-like substrates by Kolar et al. [10], after cleavage of methylated procyanidin in a D_2_O-dioxane solvent mixture. It was also reported that specifically sites 6 and 8 of ring A of pelargonidin and malvidin undergo the same exchange process in acidified D_2_O and/or acidified CD_3_OD, apparently following a keto-enol tautomerism pathway, while sites in other rings remain unlabelled [11,12,13]. 

The reactivity of ring A can be chemically related to that of phloroglucinol (1,3,5-trihydroxybenzene), which is well-known to undergo both O- and C- alkylation indicating an aromatic or carbonylic structure of phloroglucinol, respectively. The position of this keto-enol equilibrium implies several tautomeric forms and species distribution depends on specific medium and reaction conditions [14,15]. Similarly, deuteration at C(6) and/or C(8) on ring A can be interpreted, in acidic media, as a first electrophilic attack of D_3_O^+^ (or of D_2_O in neutral conditions) on activated sites of the ring A aromatic structure or as the result of solvent-mediated acid-base exchange at the α-carbonyl positions of the ring A keto structure. Totlani et al. [16] suggested that catechin-like substrates could essentially react via electrophilic aromatic substitution leading to H/D exchange [17,18]. Moreover, the two active sites (6 and 8) on ring A have been proven to have different behaviours towards H/D exchange. Other reports have found different rates of exchange for the two sites [12,19,20], which can reflect differences in their electron density, triggered by the acidity of the hydroxyl groups in position 5 and 7. Nam et al. [21] demonstrated that flavonoids undergo an exchange reaction in deuterated protic solvents (such as CD_3_OD or D_2_O) under acidic conditions. This reaction occurs much more slowly under neutral conditions as observed by following the reaction pathway during storage of the samples at −20 °C over a period of 12 months [21]. These results suggested the existence of different intermediate species (mono-deuterated species) in addition to the initial substrate and di-deuterated product. Moreover, other studies converged on the same main outcome leading to different rates for the process at the two sites C(6) and C(8), indicating a quite different electron density on them [22]. 

However, since detailed kinetic data on this H/D exchange process for flavanols in pure D_2_O at neutral pH have never been reported, we decided to exploit the NMR technique to obtain robust kinetic measurements that would allow us, in turn, not only to reliably assess the kinetic parameters of the process but also to shed light on the mechanistic details of the process. 

## 2. Results and Discussion

### 2.1. H/D Isotopic Exchange on Ring-A Protons at Positions C(6) and C(8) of Flavanols

**Scheme 2:**
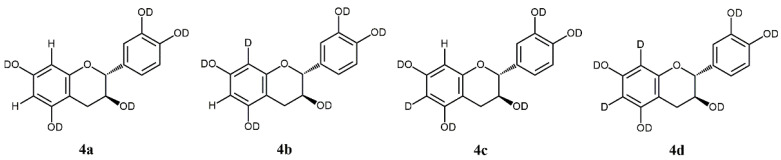
Catechin derivatives: O-D labelled (**4a**), mono-deuterated at C-8 (**4b**), mono-deuterated at C-6 (**4c**), and di-deuterated at C-6 and C-8 (**4d**). Similar structures for epicatechin-derivatives are named **5a**–**d**.

Although the solubility in water of simple flavanols, such as catechin (**1**) or epicatechin (**2**) is low (≈10 mM), aqueous homogeneous solutions can be prepared and investigated through NMR without any apparent phase separation along several days. When they are dissolved in deuterated water (D_2_O), all phenolic O-H protons are quickly exchanged by deuterium ions leading to **4a** or **5a** O-D labelled substrates (Scheme 2). At the concentrations of the homogenous solutions of **1** and **2** achievable in D_2_O (≈10 mM) the pH was measured to be 6.9 (pD = 7.3) using ^1^H-NMR signals of imidazole as pH indicator [23]. We found that catechin **4a** undergoes a slow process of deuterium incorporation leading to the fully deuterated ring A species **4d** through the partially deuterated intermediates **4b-4c**. Similarly, the O-D labelled epicatechin species **5a** in D_2_O is slowly converted into **5d**, through the intermediates **5b**-**5c**. 

Although the standard Gibbs free energy difference (∆G^0^) of the overall equilibrium process (1):

(1) $$4a+2D_{2}O\;⇄4d+2\mathrm{HDO}$$

is expected to be about zero due to self-compensating enthalpic and entropic effects, the equilibrium position is completely shifted towards the products since D_2_O is present in the reacting system in a much larger molar excess than **4a**, thus leading to complete irreversible conversion of **4a** into **4d**. Since the free energy of activation (∆G^‡^) of the process (1) is not so high as it would be expected for any C-H/C-D exchange, its rate can be evaluated at r.t. (or within a small T range around it) by following the time (t) and temperature (T) dependence of the ^1^H-NMR signals of ring A aromatic protons. 

**Figure 1:**
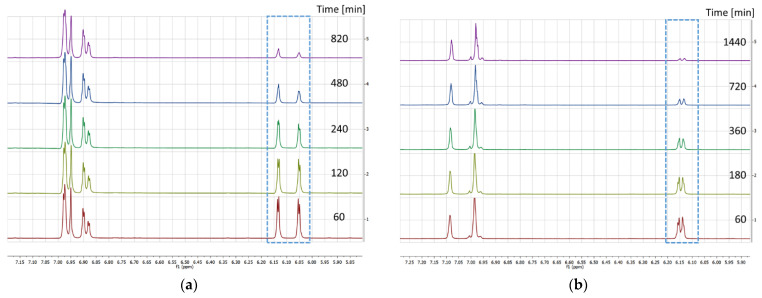
Downfield region of ^1^H-NMR spectra of 10 mM solutions of (**a**) **4a** and (**b**) **5a** in D_2_O taken at different times (T = 310 K).

In fact, at T = 310 K, **4a** was observed to undergo in a few hours a significant D-labelling at C(6) and C(8) as highlighted in Figure 1a (blue dotted boxes) by the significant decrease of the ^1^H-NMR signals H-C(6) (δ_H6_ = 6.13) and H-C(8) (δ_H8_ = 6.05). The same behaviour was shown (Figure 1b, blue dotted box) by the corresponding signals of epicatechin (**5a**) (δ_H6_ = 6.15 and δ_H8_ = 6.13).

**Figure 2:**
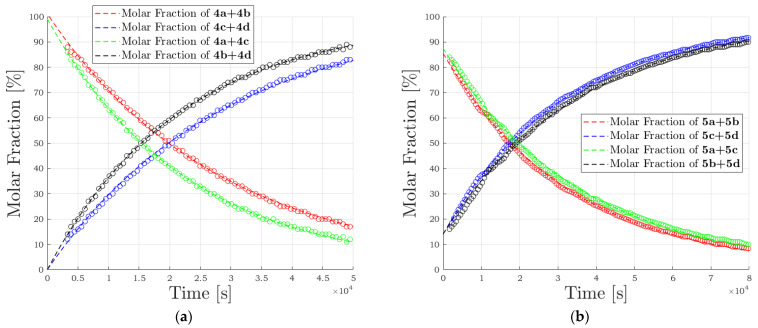
^1^H-NMR kinetics data at T = 310 K for the deuteration of (**a**) catechin in D_2_O (**4a→4d**) and (**b**) epicatechin in D_2_O (**5a** → **5d**); red-blue points refer to C(8) deuteration whilst green-black points refer to C(6) deuteration.

For the overall process **4a** → **4d** (Figure 2a) the time-varying amount of (**4a** + **4b**) and (**4a** + **4c**) were obtained from the NMR area integration of the signals H-C(6) and H-C(8), respectively, and were normalized to the unperturbed area of the ring B protons. 

By integrating the area of these signals, we evaluated not only the time dependence of the disappearance of **4a** but also of the appearance/disappearance of the monodeuterated D8 (**4b**) and the monodeuterated D6 intermediates (**4c**). 

**Figure 3:**
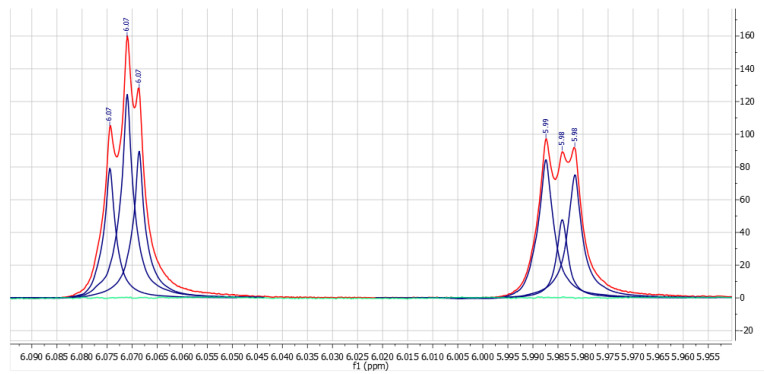
^1^H-NMR spectral deconvolution (GSD) of peaks corresponding to sites C(6) (6.07 ppm) and C(8) (5.98 ppm). ^1^H-NMR spectrum taken at t ~ 500 min from **4a** kinetics experiment at T = 305 K. Red, blue, and green curves represent the experimental ^1^H-NMR, deconvoluted peaks, and residuals, respectively.

In fact, at a given time of any kinetics run (Figure 3, t = 500 min, T = 305 K), the peak deconvolution of the signal of H-C(6) at δ_H_ = 6.07 allows establishing the relative contributions of the residual H-C(6) of **4a** (doublet, *J* = 2.1 Hz) and a new singlet signal (centred at almost the same frequency as the former) attributable to D-8 monodeuterated species **4b**.

By normalizing to the initial concentration of **4a**, the % molar fraction of these two species (x**_4a_** + x**_4b_**) was evaluated to be 60% and peak deconvolution leads to x**_4a_** ≈ 2x**_4b_**.

Correspondingly, the peak deconvolution of the signal of H-C(8) at δ_H_ = 5.98 allows establishing the relative contribution of the residual H-C(8) of **4a** (doublet, *J* = 2.1 Hz) and a new singlet signal attributable to **4c**. The % molar fraction of these two species (x**_4a_** + x**_4c_** = 52%) can be obtained by the relative area integration, whilst the peak deconvolution leads to x**_4a_** ≈ 4x**_4c_**. 

**Figure 4:**
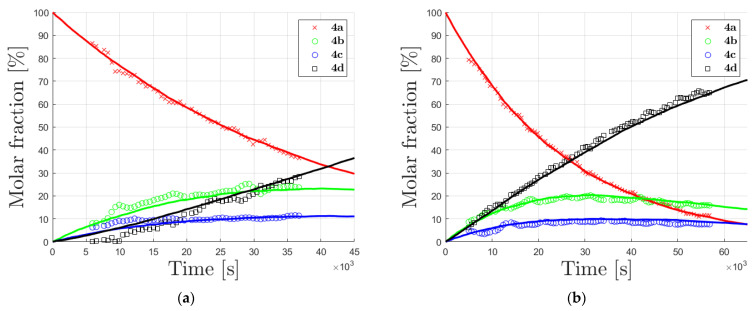
Experimental kinetic data and best fitting curves for the deuteration processes of **4a** (**a**) at T = 300 and (**b**) at T = 305 K.

To fulfil the mass balance constrain, the overall normalized % distribution was evaluated to be x**_4a_**:x**_4b_**:x**_4c_**:x**_4d_** = 42:20:1:28, indicating different specific rate constants (i.e., regioselectivity) at the two different sites with a faster H/D exchange at C(8) than at C(6). In principle, the relative amount of **4a**–**4d** species can be evaluated at any time and any fixed temperature during the kinetic run. Actually, several ^1^H-NMR spectra taken in kinetic runs carried out at higher temperature do not have sufficient resolution to allow an adequate deconvolution of H(6) and H(8) signals, but this outcome did not hinder our analysis so much. The peak deconvolution of the H-C(6) and H-C(8) signals allowed us to separate the time-dependent contribution of the intermediates **4b** and **4c**, reaching maximum values of x**_4b_** ≈ 0.20 and x**_4c_** ≈ 0.10 at about the same time (Figure 4a t_max_ ≈ 500 min). More importantly, a single exponential fitting (Figure 4) of time, the varying x**_4a_** (t), was obtained with an observed rate constant (T = 305 K) k_obs_ (**4a**) = 3.90 × 10^−5^ s^−1^ (t_½_ (**4a**) = 17,800 s) suggesting that all the processes contributing to the disappearance of **4a** are (pseudo) first-order processes, only depending on the initial concentration of **4a** (Figure 4b). 

Similar data were also obtained for the **5a** → **5d** process. However, deuteration of aromatic protons of **5a** in D_2_O (Figure 2b) does not show any site selectivity. In fact, the overall rate of disappearance of the H-6 signals (**5a** + **5b**) was almost the same as that of the H-8 signals (**5a** + **5c**). In this case, we were not able to evaluate at a given time point the relative contribution of monodeuterated **5b** and **5c** by peak deconvolution since from one side the difference of chemical shift between H-C(6) and H-C(8) in epicatechin is so small to become comparable to the *J*(6,8) coupling constant (leading to non-first order NMR signals) and, from the other side, the signal lineshape was not good enough to allow reliable fittings. 

**Scheme 3:**
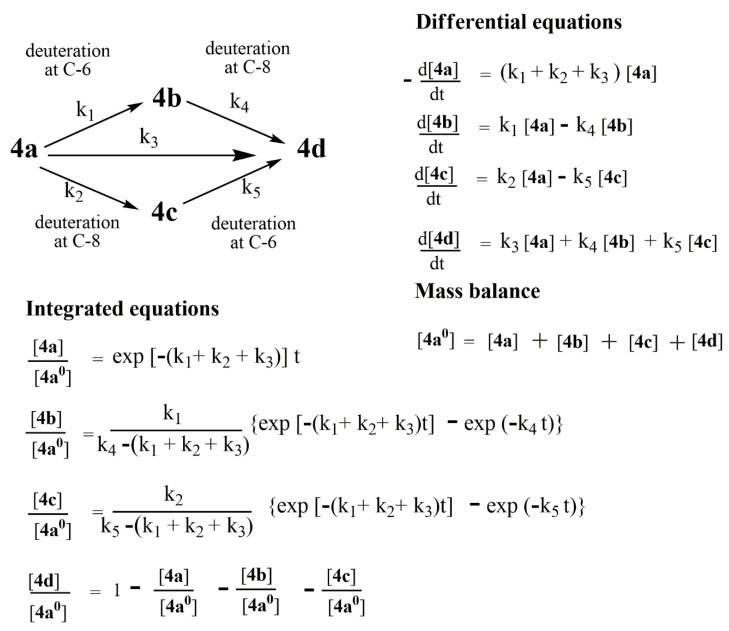
Reaction scheme of the five steps involved in the deuteration of catechin by D_2_O (top, left); differential (top, right) and integrated (bottom) kinetic laws assuming pseudo-first-order processes.

Initially, we thought that the kinetic behaviour showed by deuteration of **4a** could be rationalized by assuming two competitive pseudo-first-order conversions starting from the same substrate (**4a** → **4b** in competition with **4a** → **4c**) followed by two convergent pseudo-first-order processes leading to the same end product (**4b** → **4d** in addition to **4c** → **4d**). To evaluate the four unknown rate constants (k_1_, k_2_, k_3_, k_4_), we resorted to the Kinetiscope software using, as the initial guess for the fitting, the values roughly estimated from our raw kinetic data shown in Figure 2. However, we soon realized that it was almost impossible to find a set of four k_i_ able to adequately fulfil our experimental x_i_(t) data. No set of four k_i_ was able to fit the general trend of these curves and to be in agreement with several significant constraints imposed by experimental measures, such as the time of the crossing points of the curve x**_4d_**(t) with that of x**_4a_**(t) (26,800 s) and/or the time of the crossing point of the curve of x**_4b_**(t) with those of x**_4a_**(t) (73,000 s), x**_4b_**(t) (42,200 s), and x**_4c_**(t) (≈80,000 s). To find an optimal fitting of our data, another process needed to come into play, i.e., a process able to convert **4a** into **4d** without any detectable intermediate. Thus, we were somehow forced by experimental findings to suppose the kinetic pathway drawn in Scheme 3, based on competitive-consecutive pseudo-first-order processes. The time dependence of all the stable species is regulated by a system of linear differential equations (Scheme 3, top) that, with the appropriate boundary conditions and the mass balance constrain, lead to the integrated solution x_i_(t) here reported (Scheme 3, bottom). 

The fitting of these integrated equations with the stochastic simulator Kinetiscope leads to the following best fitting values at T = 305 K: k_1_ = 1.7 × 10^−5^ s^−1^, k_2_ = 0.85 × 10^−5^ s^−1^, k_3_ = 1.35 × 10^−5^ s^−1^, k_4_ = 2.5 × 10^−5^ s^−1^, and k_5_ = 2.4 × 10^−5^ s^−1^, fulfilling the condition k_obs_ = (k_1_ + k_2_+ k_3_) = 3.90 × 10^−5^ s^−1^. By introducing these values into integrated equations, we found that the calculated curves were almost superimposable to our experimental kinetic data (Figure 4b). 

Since, in principle, the molar fraction of every species can be evaluated at every time (and temperature), we carried out a detailed kinetic analysis following the time dependence of the NMR-sensitive species **4a**/**4b**/**4c** performing ^1^H-NMR measurements at five different temperatures (300–320 K). Unfortunately, the peak deconvolution of the H-C(6) and H-C(8) signals was possible only at 300 (Figure 4a) and 305 K (Figure 4b) since at higher temperatures the peak resolution of these signals was not sufficient to allow reproducible deconvolutions. 

**Figure 5:**
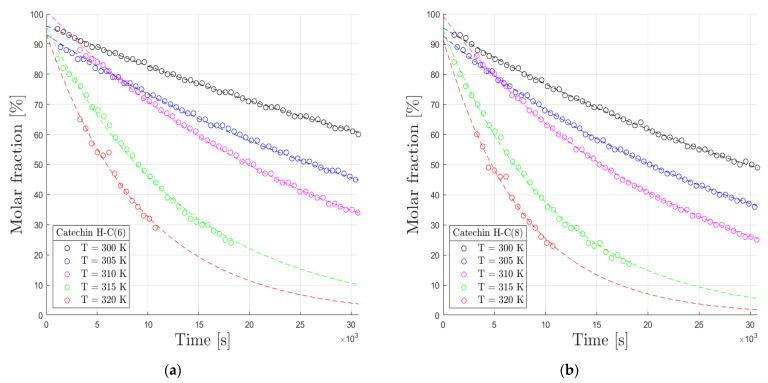
^1^H-NMR kinetic data, taken at five different temperatures, for H/D exchange at (**a**) C(6) and (**b**) C(8) in the catechin/D_2_O reacting system. Here, the site-specific decreasing H-C(6) and H-C(8) signals are plotted in order to obtain k’_obs_; although there is no simple relation between these observed rate constants and k_i_ (i = 1, 2, 3, 4, 5), these data provide useful information on the site-specific deuteration kinetics.

Thus, the kinetic parameters reported in Table 1 have just an empirical significance, representing the observed time dependence (k’_obs_) of the peak area of the H-C(6) (Figure 5a) and H-C(8) (Figure 5b) at fixed temperatures and not the true rate constants controlling the time changes of x**_4a_**(t). In other words, k’_obs_ (and the corresponding activation energies) are not simply related to the true values of the three processes, whereby **4a** is converted to the intermediates **4b** and **4c** and/or to the final product **4d**. Anyhow, these empirical data are quite useful since they allow us to estimate how and how much the average site selectivity of deuteration depends on temperature. If we define the ratio (k’_(C6)_ − k’_(C8)_)/(k’_(C6)_ + k’_(C8)_) at a given temperature as an empirical descriptor of this selectivity, we can notice that it significantly decreases by increasing the temperature, ranging from ≈0.2 (T = 300 K) until ≈0.1 (T = 320 K). This is largely expected since increasing T, the differential exponential factors regulating the rate constants ΔΔG^‡^/RT (ΔΔG^‡^ = ΔG^‡^_(C6)_ − ΔG^‡^_(C8)_) must decrease.

It is worth noting that the average ΔG^‡^ ≈ 100 kJmol^−1^ of the two competitive processes is quite similar and dominated by the contribution of the enthalpic term (75–80%). As already pointed out, however, these values simply represent the time dependence of the deuterium ion attack both to **4a** and **4b** for the H/D exchange at C(8) and both to **4a** and **4c** for the H/D exchange at C(6). While in the early part the contributions of **4b** and **4c** can be neglected, this is not true in the middle or late steps of these kinetic runs. 

A deeper insight into the mechanistic details of the pathway proposed in Scheme 3 can be achieved by considering the kinetic data for all the elementary steps obtained at two different temperatures, 300 and 305 K. For the former, the fitting of the curve x**_4a_** (t) leads to k_obs_ (**4a**, T = 300 K) = 2.71 × 10^−5^ s^−1^ (t_½_ (**4a**) = 25,000 s), with a 30% decrease with respect to the value evaluated at T = 305 K. The simulation of all the experimental evaluated x_i_(t) affords the following best fitting values at 300 K: k_1_ = 1.5 × 10^−5^ s^−1^, k_2_ = 0.75 × 10^−5^ s^−1^_,_ k_3_ = 0.45 × 10^−5^ s^−1^, k_4_ = 2.2 × 10^−5^ s^−1^, and k_5_ = 2.2 × 10^−5^ s^−1^ (Figure 4a). 

Thus, k_3_ decreases with the decrease of temperature significantly more than the other four rate constants pointing out that **4a** undergoes a different molecular rearrangement before leading the end-product **4d.** By inserting these k_i_ values into the Eyring equation we obtained the corresponding ΔG^‡^, although we cannot estimate the relative contribution of enthalpic and entropic activation terms. 

**Figure 6:**
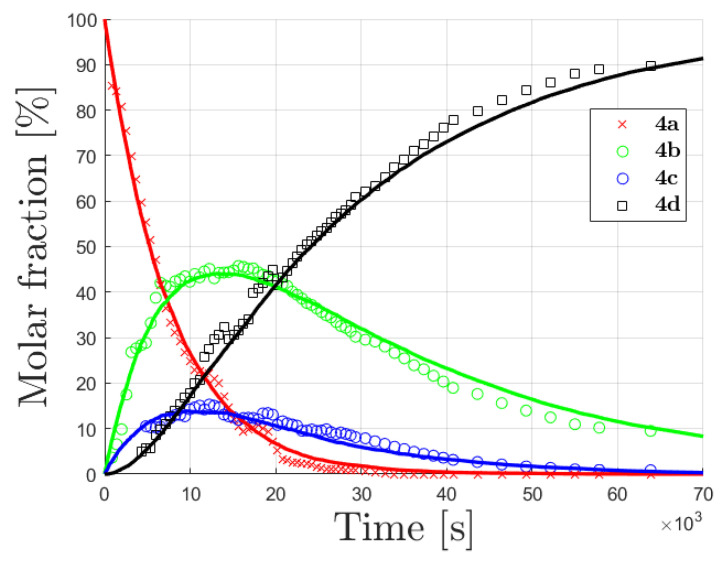
Experimental kinetic data and best-fitting curves obtained by the rate constants optimization for the deuteration process occurring on catechin at T = 300 K in D_2_O with 55 mM of acetic acid (CD_3_COOD), resulting in the calculated pH of 3.0.

Another interesting feature is the pH-dependence of these rate constants. By lowering the pH (addition of CD_3_COOD) in the **4a**/D_2_O reacting system, we observed as expected that the rate of the deuteration process increases. Thus, whilst the half-lifetime of **4a** at 300 K in neutral solution was found at about 25 min, it decreased at about 6 min at pH 3.0. However, we noticed that k_3_ became significantly lower than observed in neutral pH conditions. At pH 3.0 (300 K), this effect is largely offset (Figure 6) by the increase of k_1_ and k_2_ leading to an observed rate constant significantly larger than in neutral conditions. Another striking feature, related to the pH-induced changes of these rate constants, is represented by the significant increase of the maximum of the curves x**_4b_**(t) and x**_4c_**(t), speaking for a longer lifetime of the key-intermediates **4b** and **4c**. 

To summarize, our explanation relies on the evidence that D_2_O is strong enough as an acid to promote deuteration by the D^+^ transfer to C(8) and strong enough as a base to accept the D^+^ ion from DO-C(7). The effect of acidic catalyst seem to rely on (i) a relevant increase of the rate of the competitive processes **4a** → **4b** and **4a** → **4c** leading to higher relative amounts of the monodeuterated intermediates **4b** and **4c**, (ii) a large decrease of the rate of the process **4a** → **4d**, and iii) an almost unperturbed site selectivity of deuterium ion attack at C(6) or C(8).

Finally, we carried out kinetic runs at 300 K on **4a** using CD_3_OD in place of D_2_O as a deuterium ion source for the H/D exchange. The observed rate constant controlling the disappearance of **4a**/CD_3_OD was found much lower than that of **4a**/D_2_O. 

### 2.2. Mechanistic Considerations

In a Frontier Molecular Orbital approach (FMO), the mechanism of the rate-determining steps can be considered as the addition of an electrophilic species (D_3_O^+^/D_2_O) to the highest nucleophilic centres of flavanols, i.e., C(6) or C(8) which are electrons enriched by the multiple *ortho* and *para* mesomeric effect of the oxygen atoms on ring A. This attack will occur at the nucleophilic site having the largest electron density. According to ^1^H and ^13^C-NMR experimental spectra of **4a**, no matter the solvent, both H-C(8) and C(8) are slightly shielded with respect to H-C(6) and C(6) confirming their higher nucleophilicity (Figure 1a). Similar data were also obtained for the **5a** → **5d** process (Figure 1b). 

The main outcomes of our kinetic data on deuteration of simple flavanols such as **4a** and **5a** in D_2_O are here summarized: (i) In a first preliminary step, all O-H bonds are deuterium ion exchanged through a very fast process; (ii) at r.t., flavanols undergo slow H/D exchange at C(6) and C(8) or at both sites; (iii) the first step of deuteration occurs at a slightly higher specific rate at C(8) than at C(6); (iv) the second step of deuteration occurs at both sites with quite similar specific rates (k_3_ ≈ k_4_), but slightly faster than the first step; (v) the average ΔG^‡^ (≈102 kJmol^−1^) of all these deuteration processes is mainly determined by enthalpic effects; (vi) in other protic, but less polar, solvents (such as CD_3_OD), these processes occur on a significantly longer time-scale. 

**Scheme 4:**
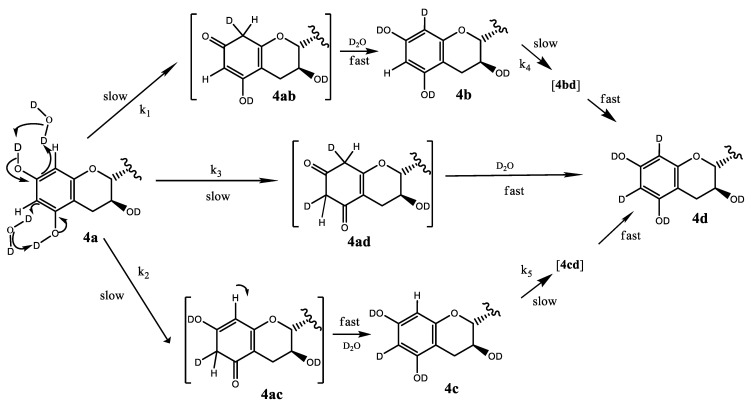
Proposed reaction mechanism for H/D exchange at C(6) and C(8) in the catechin/D_2_O reacting system.

With this kinetic information, we can propose a reasonable reaction mechanism (Scheme 4). It relies on sequential elementary steps, involving a slow, solvent-mediated, C-D bond formation/O-D bond-breaking followed by a fast, solvent-mediated, C-H bond breaking/O-D bond formation where ring-A can regain its aromaticity. Although our observed rate constants (k_i_, i = 1, 2, 3, 4, 5) and the corresponding ΔG^‡^ values are not related to true elementary reaction steps (i.e., involving single transition states), they can be considered a simple sequence of elementary steps with one of them as the rate-determining step. Assuming the well-known and largely accepted mechanism involving keto-enol tautomerism of 1,3,5 activated aromatic systems [24], we propose that deuterated keto-analogues (**4ab** and **4ac**) could be the putative transient intermediates leading to the detectable intermediates **4b** (k_1_) and **4c** (k_2_) in the two competitive divergent processes **4a** → **4b** and **4a** → **4c**, respectively. Similarly, **4bd** and **4cd** could be the transient putative intermediates of the two convergent pathways, **4b** → **4d** regulated by k_4_ and **4c** → **4d** regulated by k_5_ leading to the end-product **4d**. However, as already discussed, our data suggest a relevant role played by the direct process **4a** → **4d**, implying the putative intermediate 5,7-diketo derivative **4ad**.

Although we can only speculate about the structures of the transition states leading to the above-cited putative intermediates, since the elementary steps dealing with C-D formation/O-D breaking and loss of aromaticity are highly endothermic, they are also expected to be the rate-limiting steps and the structure/energy of the relevant TS similar to the structure/energy of the putative intermediates. 

The plausibility of the termolecular elementary process **4a** → **4d** relies on the need to introduce another step converting **4a** into **4d** in addition to the bimolecular elementary steps of mono-deuteration. Practically speaking, this termolecular process is feasible due to the interaction between three molecules (**4a** + 2D_2_O) and could be energetically comparable (if not favourable) over the sequential competing bimolecular mono-deuteration processes [22], leading to lower enthalpic contribution (ΔH^‡^) of the step **4a** → **4ad** (assuming within the Hammond postulate that the corresponding transition state has a structure similar to **4ad**), only partially counterbalanced by a greater entropic term contribution (−TΔS^‡^), in agreement with the lower probability to arrange two D_2_O molecules in the correct positions for the H/D exchange event. 

The relevant outcome that deuteration processes of **4a** occur much more slowly in CD_3_OD than in D_2_O [25] can be explained in our view by the proton/deuterium acid-base properties of flavanols in methanol (pK_a_ ≈ 13–14) with respect to water (pK_a_ ≈ 9–10) [26]. The lower acidity of the O-H(D) groups in methanol reflects the reduced thermodynamic ability of methanol to solvate the ions produced by the proton-transfer reaction. However, since the loss of proton/deuterium ions is also involved in all the transition states leading to the putative intermediates, we believe that it can play a relevant role also in the kinetics.

### 2.3. H/D Isotopic Exchange on Ring-A Proton at Position C(8) of Quercetin

**Figure 7:**
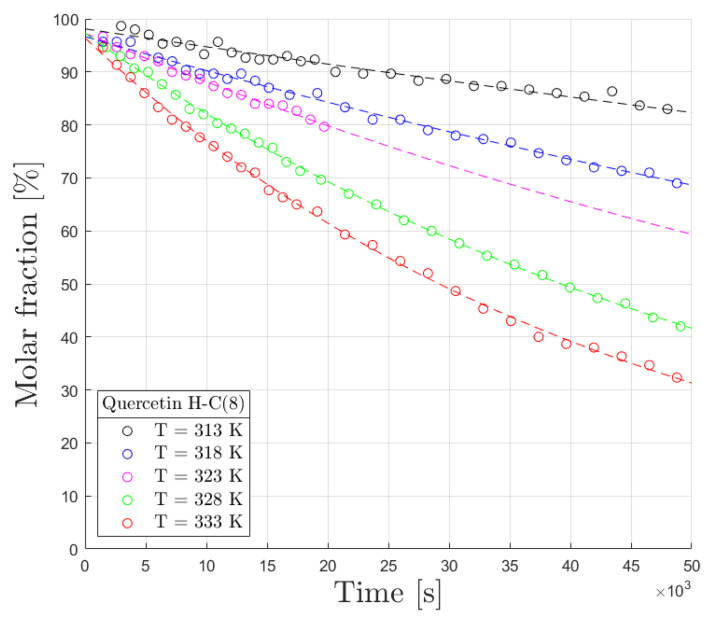
^1^H-NMR kinetics data for H/D exchange at C(8) by D_2_O at five different temperatures in quercetin **3** in D_2_O/DMSO_d6_ 1:1 solution.

The solubility of quercetin (**3**) in water is so low (≈3 mM at 300 K) [27] to hinder the NMR measurement of deuterium ion incorporation by D_2_O, which is expected to be slower than in flavanols. However, in D_2_O/d_6_-DMSO 1:1 solution, the solubility can be enhanced and 10 mM solutions of quercetin in this solvent mixture were appropriate to carry out a full kinetic analysis in the temperature range T = 313–333 K. The 4-keto group on ring B strongly affects the electron density on ring-A positions, in particular at C(6) position, in as much that only deuteration at C(8) can be observed in our conditions. Following the similar approach described above for flavanols, we were able to evaluate the Gibbs free energies of activation (Figure 7) for H/D exchange at C(8) in **3** by running the same reaction at different temperatures (Table 2). 

The deuteration rate constants for **3** are about a magnitude order lower (and the corresponding ΔG^‡^ values about 10 kJ/mol higher) than those discussed above for **4** and **5**. In our hands, deuteration at C(6) occurs at a so low rate to be considered negligible. Findings by Faizi et al. [9] of C(6) deuteration for **3** quercetin in CF_3_COOD are not in contrast with our results, since in these acidic conditions deuteration at C(8) is so fast that at the start of the analysis it was already completely exchanged.

### 2.4. Results from ab Initio DFT Calculations 

To get further molecular details on our kinetic investigations, we resorted to density functional theory (DFT) calculations through codes implemented in the Gaussian 16 software [27]. To arrive at the energy minima of all the structures of flavanols here reported, a full conformational search with the aid of the GMMX add-on (GaussView program) was undertaken. Conformational searching was carried out, allowing both ring C flipping and single-bond rotations through the use of the default values of the program. The MM-minimized rotamers were used as initial input structures, and the geometry was energy-minimized using the DFT/B3LYP 6–311G level of theory [28].

**Figure 8:**
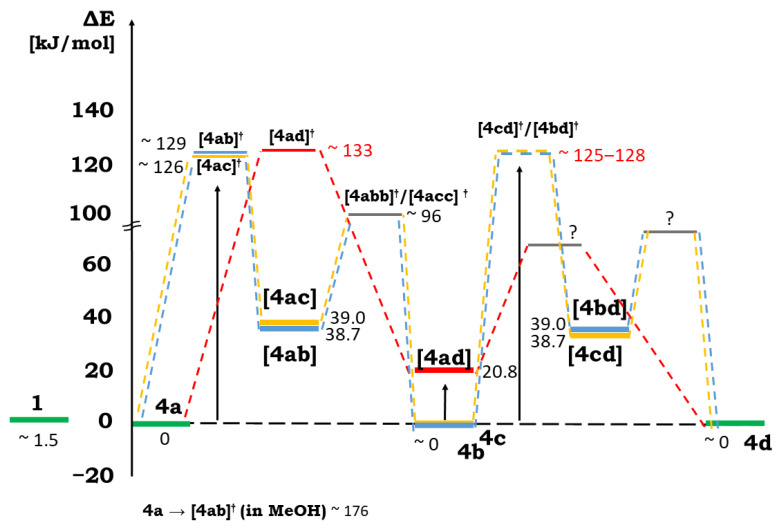
Energy profile visualization of the putative intermediates and transition states involved in catechin/D_2_O deuteration process **1** → **4a** → **4d**.

Regarding the catechin system, calculations carried out at the B3LYP/6-311G level of theory confirmed the higher Mulliken negative charge at C(8) (−0.20) with respect to that at C(6) (−0.17). Then, ab initio calculations (geometry optimization followed by frequency calculations) were used to estimate the relative energies of all the species participating in this pathway. A set of simulations were also performed to optimize structures and energies of the corresponding transition states. The calculated energy profile is summarized in Figure 8.

Energies of the mono- and di-deuterated keto forms **4ab**, **4ac**, **4ad**, **4bd**, and **4cd** were evaluated by QM calculations to be significantly higher than the corresponding mono- and di-deuterated enol forms **4a**, **4b**, **4c**, and **4d**. Importantly, the intermediate **4ad** was found more stable (by 16 kJmol^−1^, even after corrections of their different thermal contributions) than the competitive intermediates **4ab** and **4ac**, thus giving further support to its existence along the pathway of the overall exchange process, as already guessed by the overall fitting of our kinetic data. The transition states (**4ab**^‡^, **4ac**^‡^, **4ad**^‡^) of the three competitive deuteration processes were found about 130 kJmol^−1^ above the energy of reactant **4a**. All the structures associated to **4ab**^‡^, **4ac**^‡^, **4ad**^‡^ species showed only one imaginary frequency thus ensuring we were dealing with true transition states. Interestingly, the calculations were also in agreement with the experimental evidence that specific rates of deuteration are lower in deuterated methanol than in deuterated water. In fact, the energy of the **4ab^‡^** analogue obtained by deuterium ion transfer from CD_3_OD was found about 35 kJmol^−1^ higher than that of **4ab^‡^** itself. The intrinsic reaction coordinate (IRC) calculation performed on **4ab^†^** (Supplementary Figure S1) afforded reactant **4a** and product **4b** showing that during the process there is a synchronous breaking of the bond D-OC(7) and formation of the bond D-C(8). 

Even the bond length O-C(7) is a fine reaction coordinate since, as expected, it undergoes a significant shortening ongoing from the enol form **4a** to the keto form **4b**.

The calculated rate constants are of course largely lower than the experimental ones (10^−10^ s^−1^ versus 10^−5^ s^−1^) since the calculated free energies of activation are about 30 kJ/mol higher than those experimentally measured. However, we are confident that, by putting two and not only one D_2_O molecules in the reactive site of these TS, their energy would become significantly lower due to the well-documented assistance [29] of the 2nd D_2_O molecule enhancing the deuterium ion donor and deuterium ion acceptor capability of the first reacting D_2_O molecule. Basically, in our approach, the energy profile reported in Figure 8 is aimed to just provide an approximate agreement (in terms of minimum energy of all species playing a role in the process, such as reactants, products, putative, and isolated intermediates) with our experimental findings. Thus, activation energies of processes **4a** → **4b** and **4a** → **4c** were calculated to be almost the same (in fact k_1_ ≈ k_2_), as well as the processes **4a** → **4b** and/or **4c** → **4d** were also calculated to have quite similar activation energies as process **4a** → **4d**, (in fact k_3_ ≈ k_1_, k_2_, k_4_, and k_5_). 

## 3. Materials and Methods

Each sample was prepared directly in the NMR glass tube. In addition, 10.0 ± 0.3 mM solutions of starting material 1 and 2 were prepared by dissolving 2 mg of pure compounds in 0.750 mL of D_2_O with the help of a sonic bath. For compound 3, a 1:1 (0.325:0.325 mL) mixture of D_2_O and DMSO_*d*6_ was necessary to solubilize the pure quercetin, without altering the reaction conditions, since D_2_O is maintained in extremely large molar excess with respect to 3. The pH of the solution was controlled directly via NMR utilizing pure imidazole ca. 0.2 mM, following the procedure described by Orgovàn et al. [23].

Kinetics of deuteration of catechin and epicatechin by D_2_O at T = 300–320 K (variable temperature unit ensure δT accuracy within ± 0.1 K) were performed using Bruker Avance 400 MHz NMR spectrometer. ^1^H-NMR measurements were carried out using a 5 mm BBI probe with a 90° proton pulse length of 8.7 μs at a transmission power of 0 dB and equipped with pulsed gradient field utility. ^1^H-NMR spectra were recorded every ~10 min and automatic peak integration of the reference signal, which maintained the same area (100%) throughout the whole experiment and of the signals of the two hydrogen atoms that are being substituted by deuterium was performed. The chemical shift scale (δ) was calibrated on the residual proton signal of trimethylsilyl-propionic-2,2,3,3-d4 acid sodium salt (TSP) that was used as reference standard (δ_H_ = 0.00 ppm).

^1^H-NMR of catechin (D_2_O, 300K): 6.86 (d, *J* = 1.9, 1H, H-2′); 6.84 (dd, *J* = 1.9, 8.1 Hz, 1H, H-6′); 6.76 (d, *J* = 8.1 Hz, 1H, H5′); 6.01 (d, *J* = 2.3, 1H, H-6); 5.91(d, *J* = 2.3, 1H, H-8); 4.60 (d, *J* = 7.9, 1H, H-2); 4.06 (ddd, *J* = 8.3, 7.9, 5.4, 1H, H-3); 2.78 (dd, *J* = 16.0, 5.4, 1H, H4a); 2.42 (dd, *J* = 16.0, 8.3, 1H, H4b). 

^1^H-NMR of epicatechin (D_2_O, 300 K); 6.86 (d, *J* = 1.9, 1H, H-2′); 6.84 (dd, *J* = 1.9, 8.1 Hz, 1H, H-6′); 6.76 (d, *J* = 8.1 Hz, 1H, H5′); 6.02 (d, *J* = 2.3, 1H, H-6); 6.00 (d, *J* = 2.3, 1H, H-8); 4.82 (brs, 1H, H-2); 4.22 (m, 1H, H-3); 2.82 (dd, *J* = 17.0, 4.3,1H, H4a); 2.67 (dd, *J* = 17.0, 2.1, 1H, H4b). 

The molar percentage ratio of the decrease of the former and increase of the latter was plotted for each compound analysed (catechin and epicatechin) and for each proton. Using Eyring and Arrhenius plots and kinetic equations, it was possible to obtain all the meaningful kinetic and energetic quantities (rate constants, half-life times, activation energy, and Gibbs free energy of activation). A qualitative separation of Gibbs free energy of activation into its two contributions was also carried out using the best fitting curve. The reaction can be considered a pseudo-first-order process since D_2_O is in large excess with respect to flavan-3-ols monomers. The same reasoning was employed also for flavonol quercetin 3 and its related kinetic analysis. The only difference for the latter is its poor solubility in pure D_2_O leading to the need for the use of D_2_O/DMSO_d6_ 1:1 solution, as solvent. 

Peak deconvolution was performed using the MestReNova v12 global spectral deconvolution tool (GSD) with default parameters. Automatic peak identification and integration were also performed to obtain molar ratios of the involved structures. Rate constants guess for best curve fitting of the reaction kinetics was performed with the help of the Kinetiscope software. Molecules’ geometry, energetics and properties simulations were performed via the Gaussian 16 suite of programs [27]. All simulations were performed with DFT at the B3LYP level of theory, at first with a 6-31G basis set, which was then expanded to 6-311G to improve results accuracy. The chemistry of these molecules and processes was modelled in the solution, using the IEFPCM continuous solvent model. Standard optimization and frequency calculations were performed on all molecules, together with transition state optimization and IRC calculation to complete the computational landscape.

## 4. Conclusions

A careful kinetic analysis of the H/D exchange processes occurring at C(6) and/or C(8) of flavanols (**1** and **2**) or flavonol (**3**) has been carried out by joining information deriving both from experimental ^1^H-NMR spectra taken at different temperatures, pH and protic solvents (D_2_O, CD_3_OD, D_2_O/DMSOd_6_), and from theoretical QM calculations (DFT, B3LYP 6-311G). For the first time, we report here on: (a) The existence, along the reaction coordinate leading from **1** (**4a**) to the 6,8 di-deuterated analogues **4d**, of the monodeuterated species **4b** and **4c**; (b) different rates of formation of **4b** and **4c** as established by the peak deconvolution of the H-C(6) and H-C(8) NMR signals during the kinetics; (c) the relevant role played by termolecular processes whereby **4a** is converted in **4d** without passing through intermediates **4b** and **4c**; (d) the complex dependence of the overall rate constant on the pH imposed to the reacting system due to the opposite pH-response of the (two steps) route (**4a** → **4b** → **4d**) with respect to the (one step) route (**4a** → **4d**). 

## Figures and Tables

**Table 1:** Kinetic data for deuterium ion incorporation process by heavy water (D_2_O) on catechin and epicatechin substrates. The values of Gibbs free energy of activation (∆G^‡^) in the case of epicatechin are theoretically calculated based on a given temperature by the Eyring equation, as the qualitative contribution division into enthalpy and entropy of activations.

Site	T [K]	k’_obs_ [s^−1^]	E_A_ [kJ/mol]	ΔH^‡^ [kJ/mol]	ΔS^‡^ [kJ/mol K]	^(1)^ ΔG^‡^ [kJ/mol]
	300	(1.48 ± 0.01) × 10^−5^				
	305	(2.42 ± 0.01) × 10^−5^				
^(2)^ C(6)	310	(3.60 ± 0.02) × 10^−5^	79.7 ± 5.5	77.2 ± 5.5	−0.081 ± 0.017	102.1 ± 7.6
	315	(6.72 ± 0.12) × 10^−5^				
	320	(10.8 ± 0.5) × 10^−5^				
	300	(2.17 ± 0.01) × 10^−5^				
	305	(3.05 ± 0.01) × 10^−5^				
^(2)^ C(8)	310	(4.49 ± 0.02) × 10^−5^	74.4 ± 9.2	71.9 ± 9.2	−0.095 ± 0.030	101.9 ± 13.1
	315	(8.91 ± 0.11) × 10^−5^				
	320	(13.1 ± 0.6) × 10^−5^				
^(3)^ C(6)	310	(2.93 ± 0.01) × 10^−5^	/	/	/	102.9
^(3)^ C(8)	310	(2.71 ± 0.01) × 10^−5^	/	/	/	103.1

^(1)^ Values calculated at T = 310 K. ^(2)^ Catechin. ^(3)^ Epicatechin.

**Table 2:** Kinetic data for H/D exchange at C(8) by D_2_O in quercetin **3** (D_2_O/DMSO_d6_ 1:1 mixture).

Site	T [K]	k’_obs_ [s^−1^]	E_A_ [kJ/mol]	ΔH^‡^ [kJ/mol]	ΔS^‡^ [kJ/mol K]	^(1)^ ΔG^‡^ [kJ/mol]
	313	(1.48 ± 0.01) × 10^−5^				
	318	(2.42 ± 0.01) × 10^−5^				
C(8)	323	(3.60 ± 0.02) ×10^−5^	80.3 ± 8.7	77.6 ± 8.7	−0.101 ± 0.027	110.3 ± 12.3
	328	(6.72 ± 0.12) × 10^−5^				
	333	(10.8 ± 0.5) × 10^−5^				

^(1)^ Values calculated at T = 323 K.

## Data Availability

Some data obtained by DFT calculations presented in this study are available in the Supplementary Materials.

## Supplementary Materials

The following are available online, Figure S1: Qualitative reaction energy profile with reactant and putative transition state and intermediate state (a). Total energy and bond length change during the steps before and after the transition state (b).

## Sample Availability

Not available.
