# Supplementary material for: H/D Exchange Processes in Flavonoids: Kinetics and Mechanistic Investigations
Source: Molecules. 2021 Jun 10;26(12):3544. doi: 10.3390/molecules26123544 (PMC8229540; doi:10.3390/molecules26123544)
Supplement: Supplementary file 1 [file molecules-26-03544-s001.zip › molecules-1210701-supplementary.pdf]

H/D exchange processes in flavonoids: kinetics and mechanistic investigations

Federico Bonaldo[1], Fulvio Mattivi[2], Daniele Catorci[1, &], Panagiotis Arapitsas[3], Graziano Guella[1, *]

[1] Bioorganic Chemistry Laboratory, Department of Physics, University of Trento, Trento, Italy; federico.bonaldo@alumni.unitn.it

[2] Department of Cellular, Computational and Integrative Biology - CIBIO and C3A, University of Trento, Trento, Italy; fulvio.mattivi@unitn.it

[3] Department of Food Quality and Nutrition, Research and Innovation Centre, Fondazione Edmund Mach (FEM), San Michele all'Adige, Italy; panagiotis.arapitsas@fmach.it

* Correspondence: graziano.guella@unitn.it

& *D. Catorci passed away on June 27th, 2020 while this manuscript was in the latest steps of preparation.*

Supplementary materials

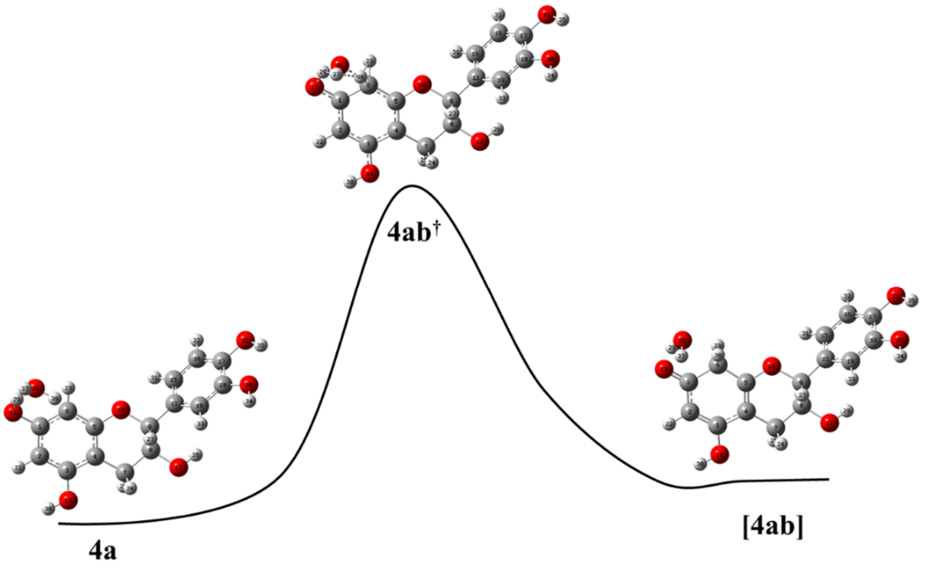

a)

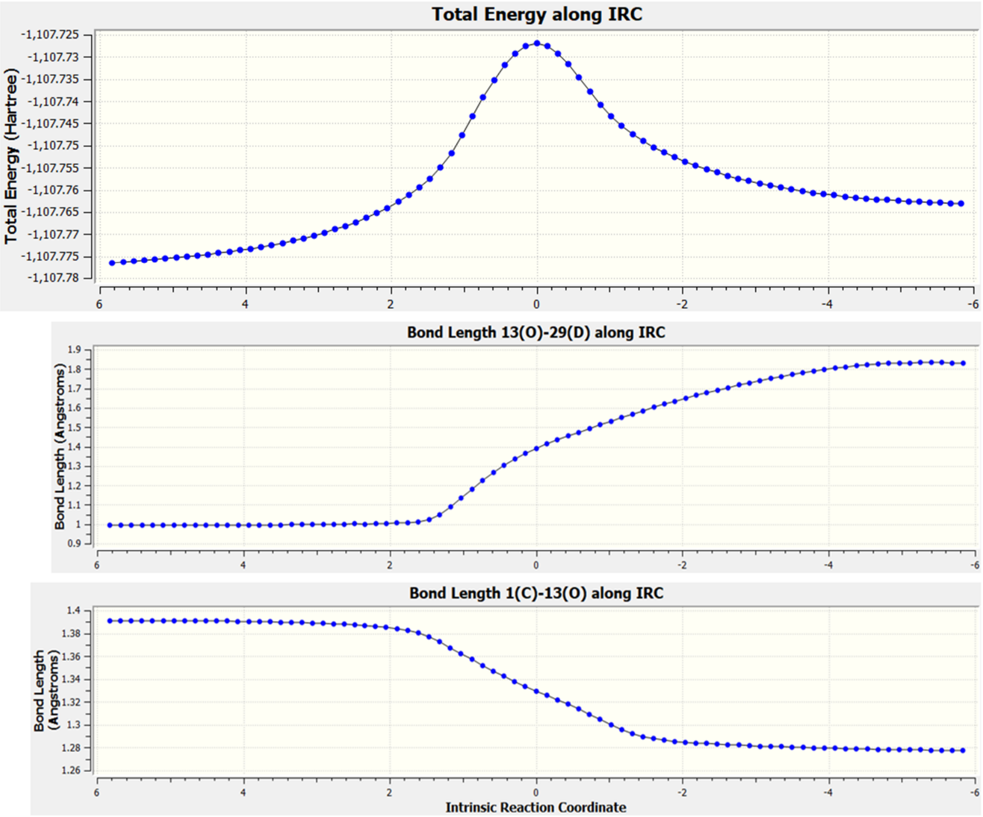

b)

Figure S1. Qualitative reaction energy profile with reactant and putative transition state and intermediate state (**a**). Total energy and bond length change during the steps before and after the transition state (**b**). Bond 13(O)-29(D) refers to bond between oxygen and deuterium in position C(7) in ring A, while 1(C)-13(O) refers to bond between oxygen and carbon at the same carbon position.
